# Optimization of Orange Peel Extract Nanoemulsions and Their Incorporation into Chitosan/Gelatin Biodegradable Films: Physicochemical and Functional Properties

**DOI:** 10.3390/polym18202533

**Published:** 2026-10-15

**Authors:** Nallely Peñuñuri-Pacheco, Nuria Muñoz-Tébar, Raquel Lucas-González, Humberto González-Ríos, Martin Valenzuela-Melendres, Juana Fernández-López, Manuel Viuda-Martos

**Affiliations:** 1 Coordinación de Tecnología de Alimentos de Origen Animal, Centro de Investigación en Alimentación y Desarrollo, Carretera Gustavo Enrique Astiazarán Rosas No. 46, La Victoria, Hermosillo 83304, Sonora, Mexico; npenunuri222@estudiantes.ciad.mx (N.P.-P.); hugory@ciad.mx (H.G.-R.); martin@ciad.mx (M.V.-M.); 2 IPOA Research Group, Institute for Agri-Food and Agri-Environmental Research and Innovation (CIAGRO-UMH), Universidad Miguel Hernández, Ctra. Beniel km 3.2, 03312 Orihuela, Alicante, Spain; nuria.mute@gmail.com (N.M.-T.); raquel.lucasg@umh.es (R.L.-G.); mviuda@umh.es (M.V.-M.)

**Keywords:** Polyphenol encapsulation, active food packaging, agro-industrial by-products, nanoemulsions, chitosan/gelatin films

## Abstract

In this study, orange peel extract (OPE), obtained from *Citrus sinensis* processing by-products, was encapsulated in nanoemulsions and incorporated into chitosan/gelatin-based biodegradable films to enhance their functional performance. A Box–Behnken design combined with response surface methodology was employed to optimize nanoemulsion formulation. Polyphenolic profiling revealed that flavanones, particularly hesperidin and narirutin, were the predominant compounds in both the extract and the optimized nanoemulsion, with retention efficiencies exceeding 80% for major flavonoids. The incorporation of OPE-loaded nanoemulsions into chitosan/gelatin films significantly modified film structure and functionality. Nanoemulsion-containing films showed increased thickness and opacity, reduced solubility and swelling capacity, as well as enhanced UV-visible light barrier properties compared with control films. Furthermore, nanoencapsulation increased polyphenol recovery within the film matrix, preserving up to 80% of total polyphenols after film formation. Both free-extract and nanoemulsion-loaded films exhibited markedly enhanced antioxidant activity compared with control films. Release studies demonstrated that nanoemulsion incorporation promoted a more sustained and controlled release of polyphenols, with the characteristic release time more than doubling relative to films containing the non-encapsulated extract. No antibacterial activity was detected against the tested bacterial strains; this result may be attributed to the relatively low availability of antimicrobial compounds in the film matrix or their limited diffusion into the surrounding medium; nevertheless, the incorporation of OPE-loaded nanoemulsions improved stability, antioxidant performance, and controlled release behavior, highlighting the potential of nanoemulsion-assisted incorporation strategies. The developed chitosan/gelatin films containing OPE-loaded nanoemulsions represent a promising approach for the development of active biodegradable food-packaging systems.

## 1. Introduction

Sustainability-related attributes and environmental labels have been reported to influence consumer preferences and willingness to pay, reflecting the increasing demand for products with lower environmental impact [1]. Modern consumer purchasing decisions are also increasingly associated with preferences for natural, transparent, and environmentally friendly products, particularly those exhibiting clean-label characteristics [2]. In response to these demands, considerable research has focused on the development of sustainable packaging systems based on biodegradable and renewable materials. Among the alternatives investigated, films and coatings produced from natural biopolymers have gained particular interest as potential substitutes for, or complements to, conventional petroleum-based plastics in food packaging applications [3,4]. These materials can be obtained from renewable resources and, depending on their composition and environmental conditions, undergo biodegradation after disposal, offering an alternative approach to reducing the persistence of plastic packaging waste [5].

Polysaccharides, proteins, and lipids have been extensively investigated as raw materials for the fabrication of biodegradable packaging materials [6,7]. Among polysaccharide-based biopolymers, chitosan has received particular interest, owing to its remarkable physicochemical, biological, and functional properties [8]. Chitosan exhibits excellent film-forming capacity, biodegradability, biocompatibility, non-toxicity, and intrinsic antimicrobial activity, making it highly attractive for food packaging applications [9]. Additionally, several studies have focused on the incorporation of bioactive compounds, reinforcing agents, and nanostructured systems to enhance the overall functionality and performance of chitosan-based packaging materials [10,11,12].

Beyond active packaging, recent research has also highlighted the potential of intelligent biodegradable packaging systems that respond to changes in the food or its surrounding microenvironment, providing information on product quality and freshness through measurable signals or visual changes [13]. These developments broaden the functionality of biodegradable packaging from passive protection towards systems capable of both interacting with the packaged food and monitoring its condition [14]. Within this context, the development of biodegradable films combining adequate structural and barrier properties with the incorporation of stabilized natural bioactive compounds represents a relevant strategy for designing active food-packaging systems, while providing a basis for further integration of intelligent functionalities for real-time quality monitoring.

Fruit-derived extracts represent one of the most promising sources of natural bioactive compounds for active food packaging applications [15,16]. Fruits and fruit processing by-products are rich in phenolic compounds, flavonoids, anthocyanins, carotenoids, tannins, vitamins, and other phytochemicals with recognized antioxidant and antimicrobial activities [17]. Oranges are one of the fruits with a high content of bioactive compounds in their composition. Particularly, orange peel, the main by-product of juice extraction, is a source of phytochemicals, mainly vitamin C and polyphenolic compounds such as hesperidin, narirutin, and diosmin, among others [18].

Thus, the incorporation of fruit extracts into biodegradable films may contribute to the development of active packaging systems capable of improving food preservation while simultaneously increasing the added value of agro-industrial by-products. Several studies have demonstrated that the incorporation of these extracts into biodegradable films can significantly enhance their antioxidant and antimicrobial properties [12,19,20]. However, the direct incorporation of natural extracts into polymeric matrices may also generate undesirable effects, including phase separation, reduced transparency, poor dispersion, and instability of sensitive bioactive compounds during processing and storage [21]. Furthermore, many phenolic compounds exhibit limited water solubility and high susceptibility to degradation caused by oxygen, light, and temperature, as well as to pH fluctuations [22].

One effective strategy to protect these compounds and enhance their incorporation, stability, and subsequent controlled release is through encapsulation technologies. Nanoemulsions have attracted increasing attention due to their unique physicochemical properties and technological versatility. However, optimizing the production of nanoemulsions is crucial for obtaining materials with appropriate functional properties for practical food packaging applications. Numerous studies have investigated the incorporation of nanoemulsions loaded with plant extracts or essential oils into biodegradable films, aiming to enhance their functional, physicochemical, and bioactive properties [4,9,10,23]. However, limited information is available regarding the systematic optimization of nanoemulsions specifically loaded with orange peel extracts and their subsequent integration into chitosan/gelatin matrices. Therefore, the aim of the present study was to optimize the production process of nanoemulsions loaded with orange peel extracts and to evaluate their incorporation into chitosan/gelatin-based films in order to assess their potential applicability in food packaging systems.

## 2. Materials and Methods

### 2.1. Raw Materials

Orange fruits (*Citrus* × *sinensis*), specifically the Navel Lane Late variety, were collected at the commercial ripening stage from the experimental field station of Miguel Hernández University in Orihuela, Spain. The samples were harvested during the winter of 2025. Briefly, the oranges were cleaned and peeled, and then the peel was lyophilized in a Christ Alpha 2–4 lyophilizer (B. Braun Biotech, Melsungen, Germany) for 36 h. After that, the lyophilized peel was ground and sieved (particle size < 210 µm), packaged under vacuum, and stored in darkness until use. Pumpkin seed oil (51.96% linoleic acid, 28.83% oleic acid, 12.32% palmitic acid, and 5.50% stearic acid) was purchased from Martinez-Lozano S.L. (Murcia, Spain), while soy protein was acquired from HSN (Albolote, Granada, Spain).

### 2.2. Orange Peel Extract Production

Sonotrode-assisted ultrasonic extraction, by means of Ultrasonicator UP400St (Hielscher, Teltow, Germany), was used to obtain an orange polyphenol-rich extract following the procedure previously reported by Lucas-González et al. [24]. The operating conditions were: probe S24d14D, solid-to-solvent ratio of 1:100 (g/mL), hydroalcoholic ethanol (80%, *v*/*v*), energy input of 43 J/mL, and amplitude of 80%. After extraction, the samples were centrifuged (7000× *g* for 5 min at 4 °C) and concentrated ten-fold under vacuum in a rotary evaporator R-300 (Büchi, Barcelona, Spain). An aliquot of concentrated extraction was filtered through a 0.45 μm nylon filter, collected in amber vials, and stored at 4 °C until HPLC analysis.

### 2.3. Box–Behnken Design

Box–Behnken design (BBD) was employed to optimize the ingredient amounts to carry out emulsion: soy protein concentration (X_1_: 0.3%, 1.65%, and 3%), pumpkin oil concentration (X_2_: 4.0%, 10.50%, and 17.00%), and salt (Sodium chloride) concentration (X_3:_ 0.0%, 0.30%, and 0.60%). Each factor was studied at three levels (−1, 0, +1). The design consisted of 15 runs (three center points). The monitored responses were particle size (Y_1_), polydispersity index (PDI) (Y_2_), ζ-potential (Y_3_), percentage of total polyphenols retention calculated as the sum of all individual phenolic compounds and the subfamilies of compounds (Y_4_), and percentage of narirutin retention (Y_5_), both quantified via HPLC-DAD.

### 2.4. Emulsion Elaboration and Nanoemulsion Production

Prior to the emulsion process, all ingredients were mixed. Two milliliters of a concentrated orange polyphenol-rich extract were evaporated to dryness in a rotary evaporator. Immediately afterward, salt, protein, oil, and water solution were added to the flask in the concentrations established in the Box–Behnken design. The mixture was vigorously agitated until the dried extract was fully solubilized within the emulsion ingredients. Subsequently, the entire content of the flask was transferred to a Falcon tube and homogenized using an IKA T25 UltraTurrax homogenizer (IKA^®^-Werke GmbH & Co., Staufen, Germany) at 18,800 rpm for 4 min. An ice bath was used during homogenization to prevent overheating of the sample. After emulsion formation, ultrasonication was applied to obtain the nanoemulsion using an UP400St ultrasonicator (Hielscher, Germany) equipped with an S24d7 probe at an energy input of 600 J/mL and 25% amplitude. Finally, the resulting nanoemulsion was stored for 24 h at 4 °C until further analysis.

### 2.5. Nanoemulsion Characterization

In order to analyze the formation of the nanoemulsions loaded with orange peel extracts, the particle size (nm), polydispersity index (PDI), and ζ-potential (mV) were determined using dynamic light scattering analysis with an instrument Zetasizer Advance Range (Malvern Panalytical, Worcestershire, UK) equipped with a laser at 633 nm and a scattering angle of θ = 173°. The analysis was performed in triplicate, each replicate lasting 120 s at 25 °C. The average values of the replicates were reported.

### 2.6. Polyphenolic Profile of Nanoemulsion

#### 2.6.1. Polyphenols Extraction

An aliquot of the nanoemulsion (2 mL) was collected and subjected to two extraction cycles: first with 80% methanol (80:20, *v*/*v*) and second with 70% acetone (70:30, *v*/*v*). Briefly, 20 mL of each respective solvent was mixed with the sample, sonicated in a sonication bath for 10 min, and centrifuged (7000× *g* for 10 min at 4 °C). The supernatants from both cycles were collected and evaporated under vacuum. The resulting extracts were resuspended in water, cleaned, and concentrated using a previously conditioned C18 cartridge. A mixture of formic acid and methanol (1:99, *v*/*v*) was employed to elute the samples. Each extract was filtered through a 0.45 μm nylon filter into amber vials prior to HPLC injection. The results were expressed as mg/L nanoemulsion.

#### 2.6.2. Polyphenolic Profile Identification and Quantification

High-performance liquid chromatography (HPLC) was used to identify and quantify polyphenolic compounds, following the conditions reported by Lucas-González et al. [25]. Briefly, a Mediterranean Sea18 column (250 × 4.6 mm, 5 μm particle size; Teknokroma, Barcelona, Spain) was coupled to the HPLC system 1200 series instrument (Agilent, Woldbronn, Germany) and operated under gradient elution. Detection was performed at 280, 325, and 360 nm. Compound identification was based on retention times and UV-Vis absorbance spectra by comparison with available standards. When authentic standards were not available, compounds were tentatively identified based on spectral characteristics and previously reported data [26,27]. Quantification and semi-quantification were performed using calibration curves prepared from available standards (Supplementary Table S1), which were injected under the same operational conditions as the samples.

The recovery index was calculated following Equation (1).

(1) $$\mathrm{Recovery}\;\mathrm{efficience}\;\left(\%\right)=\frac{C_{\mathrm{recovered}}}{C_{\mathrm{initial}}}\times 100$$

where C*_recovered_* is the concentration of the compound obtained after the extraction procedure, and C*_initial_* is the initial concentration of the compound present in the sample.

### 2.7. Film Elaboration

Orange extract or orange nanoemulsion-added chitosan/gelatin-based functional films were produced using the solution casting method following the recommendations of Tessaro et al. [28] with minor modifications. For the elaboration of film-forming solution, chitosan (20 g) was dissolved in 1 L of lactic acid solution (1%, *v*/*v*/) at 25 °C under continuous agitation for 24 h. On the other hand, gelatin (40 g) was dissolved in 1 L of water at 60 °C with slight stirring. Then, both chitosan and gelatin biopolymer solutions were mixed in an 8:2 ratio and stirred for 1 h to ensure homogeneous blending. After that, glycerol (35% *w*/*w* based on biopolymer) was added as a plasticizer and stirred at room temperature for an additional 1 h. This procedure was used to produce a control chitosan/gelatin film (CGF-C).

To prepare the chitosan/gelatin films incorporated with orange peel extract or orange peel extract nanoemulsions, chitosan was first dissolved in a lactic acid solution (1% *v*/*v*). The resulting chitosan solution was then combined with gelatin to obtain the chitosan/gelatin film-forming solution. Subsequently, the orange peel extract (CGF-Ex) or orange peel extract nanoemulsion (CGF-N) was incorporated into the film-forming solution at the concentrations established for each formulation. Thus, the three film-forming solutions assayed (35 mL each) were poured into sterile Petri dishes (9 cm in diameter). All samples were dried in an oven at 37 °C for 48 h. The dried films were then cooled, peeled off, and stored in a desiccator at 25 °C and 53% relative humidity. The prepared films were kept in the desiccator for no longer than four days before completing all analyses.

### 2.8. Physicochemical Properties of Films

#### 2.8.1. Thickness

The thickness (mm) was assessed using a digital micrometer C112XBS (Mitutoyo, Kawasaki, Japan) with a measurement accuracy of 0.001 mm. The measurement was carried out arbitrarily at 5 different points (4 in the periphery and 1 in the interior), as mentioned by Sarraf-Ov et al. [7].

#### 2.8.2. Moisture, Swelling and Solubility Indexes

The different samples were cut into 2 × 2 cm, weighed, and then placed in an oven at 105 °C until a constant weight was reached. The moisture values were expressed as moisture content (%). Swelling and water solubility indexes were analyzed following the methodology described by Wong et al. [29].

#### 2.8.3. Water Vapor Permeability (WVP)

The water vapor permeability of the films was determined according to the ASTM E96/E96M-16 standard [30] methodology. For CGF-C, CGF-Ex, and CGF-N films, circles (55 mm diameter) were manually sealed, stuck onto the top of the permeation cells (1.22 × 10^−2^ m^2^) containing 50 mL of distilled water, placed inside a desiccator with silica gel (0% HR, 24 °C) and weighed during 10 h at 2 h intervals. Finally, water vapor permeability (WVP) of chia films was calculated with Equation (2)

(2) $$\mathrm{WVP}\;(g\cdot \;s^{-1}\cdot m^{-1}\cdot {\mathrm{Pa}}^{-1})=\frac{W\cdot \;L}{A\cdot \;t\cdot \;∆P}$$

where w is the weight of the water that permeated through the film (g), L is the film thickness (m), A is the permeation area of the cell (m^2^), t is the time of permeation (s) and ΔP is the water vapor partial difference (Pa) across the two sides of the film.

#### 2.8.4. Color, Opacity and Light Transmittance

The color (coordinates CIEL*a*b*) of the films was measured in the reflection mode with a CM-700d spectro-colorimeter (Konica Minolta, Osaka, Japan) equipped with a CR-A33a cone, a D65 illuminant and a 10° standard observer. The color-coordinated lightness (L*), redness (a*) and yellowness (b*) were measured in triplicate.

The opacity of the films was calculated in triplicate using Equation (3):

(3) $$\mathrm{Opacity}\;\left(\%\right)=\;\frac{\left(Y_{b}-Y_{w}\right)}{Y_{b}}\;\times \;100$$

where Y_b_ is the opacity on a black standard, and Y_w_ is the opacity on a white standard. The light transmittance of chitosan/gelatin films was determined according to the methodology described by Dick et al. [31]. Chitosan/gelatin film samples (3 cm × 0.9 cm) were placed in a spectrophotometer cell, and transmittance was assessed by spectrum scanning (wavelengths from 200 to 900 nm) with a UV-Vis spectrophotometer UV-5100B (Hinotek, Ningbo, China). Air was used as a reference, and the transmittance values (expressed as % of transmittance) were measured in triplicate.

### 2.9. Polyphenolic Profile of Films

The extraction, quantification and identification of polyphenolic compounds from the CGF-C, CGF-Ex, and CGF-N films were carried out using the entire film sample, following the procedure described in Section 2.6. The results were expressed as mg/g film.

### 2.10. Antioxidant Activity

The antioxidant activity of the different film samples (CGF-C, CGF-EX, and CGF-N) was determined by using two different methodologies: 2,2′-diphenyl-1-picrylhydrazyl radical scavenging assay (DPPH) as described by Brand-Williams et al. [32] and the Ferric Reducing Antioxidant Power (FRAP) assay, which was measured according to the methodology defined by Oyaizu [33]. The extracts analyzed were those obtained as mentioned in Section 2.6.1. The results were expressed as µg Trolox Equivalents/g film.

### 2.11. Antimicrobial Activity

#### 2.11.1. Microorganisms 

The antibacterial properties of CGF-C, CGF-Ex and CGF-N films were assessed against the following bacterial strains: *Listeria innocua* CECT 910, *Bacillus* sp. CECT 40, *Serratia marcenscens* CECT 854, and *Escherichia coli* B, CECT 101. These species were supplied by the Spanish Type Culture Collection (CECT) of the University of Valencia. These bacteria were chosen because they are commonly associated with refrigerated foods as indicators of pathogenic bacteria or as spoilage bacteria.

The strains were stored in cryovials containing 15% (*v*/*v*) glycerol and maintained at −80 °C until use. For experimental assays, active cultures were prepared in sterile Mueller–Hinton broth (MHB) (Biokar Diagnostics, Beauvais, France) and incubated at the optimal growth temperature for each microorganism until the exponential growth phase was reached. Subsequently, aliquots of each culture were transferred into fresh MHB and incubated overnight under the same growth conditions. Culture purity and cell concentration were verified by plate observation and colony counting.

#### 2.11.2. Culture Conditions

The agar disc diffusion method described by Tepe et al. [34], with some modifications, was used to determine the antibacterial capacity of CGF-C, CGF-Ex and CGF-N films.

Briefly, 100 μL of a bacterial suspension adjusted to 10^6^ CFU/mL for *E. coli* and *S. marcescens* or 10^8^ CFU/mL for *L. innocua* and *B.* sp. was spread onto solid medium plates containing Nutrient Agar II (Oxoid, Basingstoke, Hampshire, England). CGF-C, CGF-Ex and CGF-N film discs, 1 cm in diameter, were aseptically obtained and placed on the inoculated plates. After that, plates were incubated at 30 °C for 24 h in the case of *Bacillus* sp., at 26 °C for 24 h in the case of *S. marcenscens*, and at 37 °C for 24 h in the case of *E. coli* and *L. innocua*. The diameters of the inhibition zones were measured in millimeters. Three independent replicates were performed on different days for each film formulation and microorganism.

### 2.12. Release Study of Films

The release of polyphenolic compounds from the studied films was carried out following the methodology described by Thi Nguyen et al. [35], with minor modifications. A 10% ethanol solution (*v*/*v*) was used as an aqueous food simulant to evaluate the release behavior of polyphenolic compounds under conditions representative of contact with predominantly aqueous food matrices (such as certain meat products). The release experiments were carried out at 4 °C for 48 h to simulate refrigerated storage conditions. The films were placed in a plastic container containing 65 mL of the food simulant. Subsequently, 500 μL aliquots were taken every 5 min during the first hour, and then at 3, 4, 24, and 48 h. The experiments were run in triplicate for each film composition. Each aliquot was filtered through a 0.45 μm nylon filter and injected into the HPLC system under the conditions described in Section 2.6.2. The total polyphenolic compounds released was calculated as the sum of the individual phenolic compounds quantified, and individual polyphenols were expressed in mg/g of film or as a percentage.

#### Mathematical Modeling of Polyphenols Release

Two mathematical models were employed to study the release behavior of phenolic compounds, as commonly reported in the literature [35]. The dissolution and mass transfer kinetics of phenolic compounds through the active films were analyzed using the Weibull empirical model (Equation (2)) [36]. This function was applied to fit the experimental release data from the initial time until the equilibrium plateau was achieved, as expressed in Equation (4).

(4) $$\frac{M_{t}}{M_{\infty }}=1-\exp(-{\frac{t}{\tau })}^{\beta }$$

where M_t_/M_∞_ is the fraction of phenolic compounds at time t; τ is the release time constant, which defines the time required to release 63.2% of the active compounds; and β is the shape parameter used to discern the predominant diffusion and polymer relaxation mechanisms.

The release kinetics were also studied using Peleg’s model [37] by fitting Equation (5) to the experimental data.

(5) $$M_{t}=\frac{t}{k_{1}+k_{2}t}$$

where M_t_ is the total phenolic content (or individual compound) released at time t, k_1_ is a constant inversely related to the initial mass transfer rate (i.e., the resistance to release at the beginning), and k_2_ is a constant related to the asymptotic value, which corresponds to the equilibrium release.

### 2.13. Statistical Analysis

The Box–Behnken design with response surface methodology (RSM) was used to optimize the emulsion formulation. The significance of the models was assessed using analysis of variance (ANOVA), along with the coefficient of determination (R^2^) and the lack-of-fit test, using Design-Expert software (version 13.0.5.64; Stat-Ease Inc., Minneapolis, MN, USA).

The associated mathematical model is expressed as follows:

$$y\;=\;\beta_{0}+\;\sum_{\left\{i=1\right\}}^{3}\beta_{i}X_{i}+\;\sum_{\left\{i=1\right\}}^{3}\beta_{\mathrm{ii}}X_{i}^{2}+\;\sum_{\left\{i<i\right\}}\beta_{\mathrm{ij}}X_{i}X_{j}\;+\;\varepsilon$$

where Y represents the dependent (response) variable, X_i_ and X_j_ denote the independent variables (coded factor levels), β_0_ is the intercept, and β_i_, β_ij_, and β_ii_ are the regression coefficients for linear, interaction, and quadratic terms, respectively. The goodness-of-fit and quality of the models were evaluated using the coefficient of determination (R^2^), the lack-of-fit *p*-value, and the coefficient of variation. The statistical significance of the models and their corresponding terms was evaluated by analysis of variance (ANOVA) at a 95% confidence level (*p* < 0.05). The 3D response surface plots were generated from the fitted models to visualize the interactions between the extraction parameters. To determine the optimal levels of the independent variables (X) for each response variable, the desirability tool within the MSR was used with the Design-Expert software. Once the predicted optimal conditions were obtained, they were tested experimentally and compared (predicted vs. experimental) to validate the prediction models. For the physicochemical, antioxidant and release properties, the results are presented as mean values accompanied by their corresponding standard deviations in the respective tables and figures. Statistical analyses were conducted using IBM SPSS Statistics software (version 26; IBM Corp., Chicago, IL, USA). Significant differences among treatments were assessed by one-way analysis of variance (ANOVA) at a 95% confidence level (*p* < 0.05). When significant effects were detected, Tukey’s post hoc test was applied to identify pairwise differences between samples.

## 3. Results and Discussion

### 3.1. Effects of Parameters Studied

Table 1 shows the results of each of the 15 experimental runs. The values ranged from 102.37 to 1944.70 nm, 0.22 to 1.00, −1.23 to −30.30 mV, 37.7 to 87.7%, and 35.6 to 94.1% for particle size, polydispersity index (PDI), ζ-potential, percentage of total polyphenol and narirutin retention, respectively. The results showed a considerable variation in particle size and PDI depending on the formulation composition. Although some formulations exhibited particle sizes within the nanoscale range, other experimental conditions resulted in substantially larger particles, exceeding 1000 nm. Similarly, PDI values ranged from 0.222 to 1.000, indicating marked differences in particle-size distribution among formulations. Therefore, the term “nanoemulsion” should be interpreted in the context of the optimized formulation rather than as an indication that all experimental conditions generated uniformly nanosized and monodisperse systems. This variability highlights the strong influence of soy protein, pumpkin oil, and salt concentrations on the physicochemical characteristics of the emulsions and supports the use of response surface methodology to identify the most favorable formulation region.

Table 2 shows the regression coefficients, coefficients of determination (R^2^), and model significance for all response variables studied. The models fitted to predict particle size, PDI, ζ-potential, total polyphenol and narirutin retention were statistically significant (*p* < 0.05). None of the models showed a significant lack of fit (*p* > 0.05), and the R^2^ values ranged from 0.50 to 0.96. The models for particle size, PDI, ζ-potential, and total polyphenol retention showed relatively high coefficients of determination, whereas the model for narirutin retention exhibited a more moderate explanatory capacity (R^2^ = 0.5005). These results indicate that the fitted models adequately described the experimental relationships between the formulation variables and the responses within the investigated design space; however, the predictive performance varied among the response variables. In particular, the relatively high variability observed among the replicated center points should be considered when interpreting the optimization results. The center-point replicates showed coefficients of variation of 43.6%, 37.3%, and 40.6% for particle size, total polyphenol retention, and narirutin retention, respectively. This variability indicates a degree of experimental uncertainty that may reduce the precision of individual predictions. Nevertheless, the absence of significant lack of fit supports the suitability of the models for describing overall response trends and identifying favorable formulation conditions within the experimental domain.

**Figure 1:**
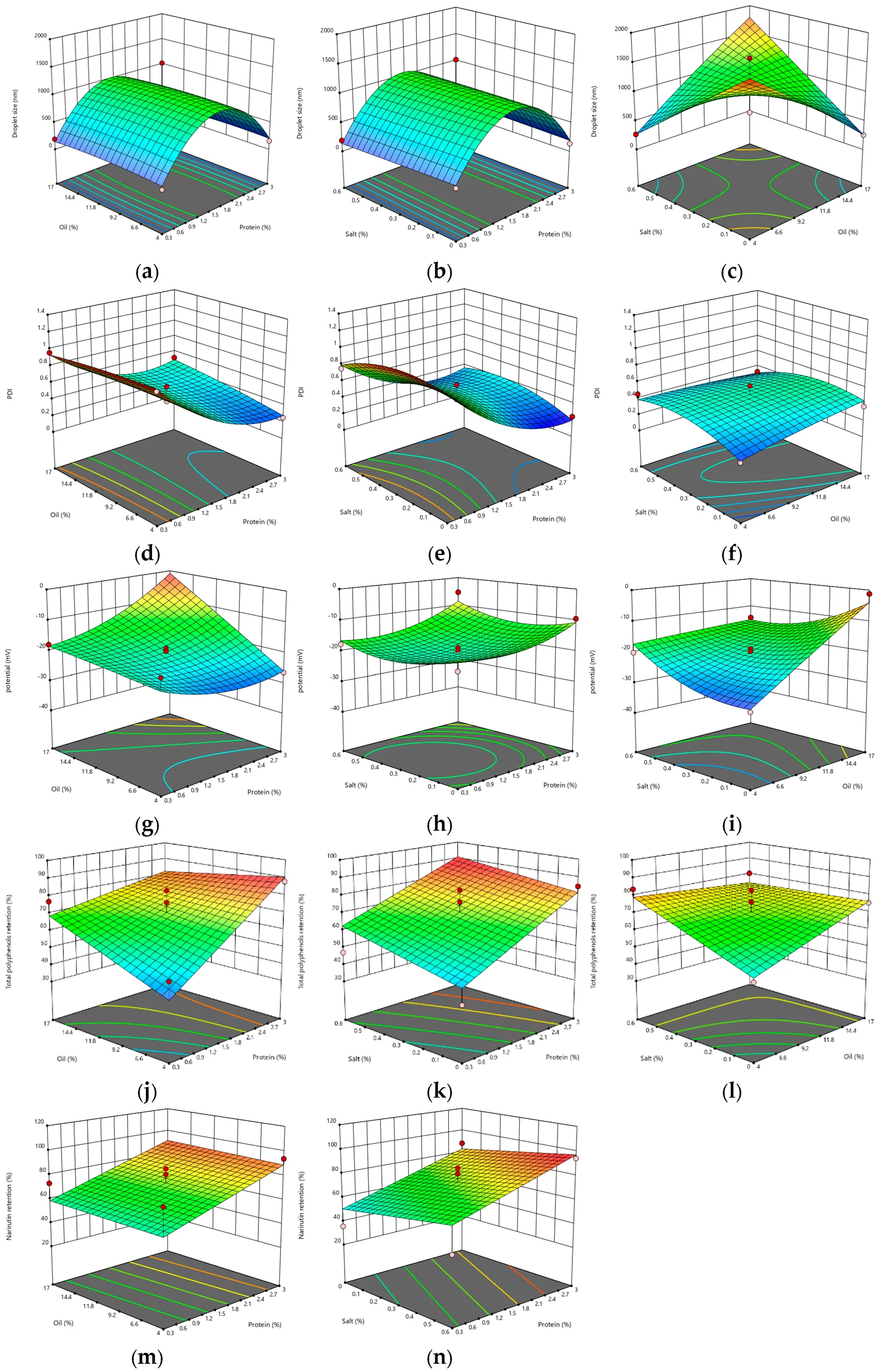
Response surface and contour plots showing the effects of protein and oil (**a**), salt and protein (**b**) and salt and oil (**c**) on droplet size; protein and oil (**d**), salt and protein (**e**) and salt and oil (**f**) on PDI; protein and oil (**g**), salt and protein (**h**) and salt and oil (**i**) on ζ potential; protein and oil (**j**), salt and protein (**k**) and salt and oil (**l**) on total polyphenol retention; protein and oil (**m**), salt and protein (**n**) on narirutin retention.

Furthermore, Figure 1 illustrates these effects as 3D plots, showing the direction of the independent variables’ effects on particle size (Figure 1a–c), PDI (Figure 1d–f), ζ-potential (Figure 1g–i), retention of total polyphenols (Figure 1j–l) and narirutin (Figure 1m,n).

Particle size, PDI, ζ-potential, and retention of total polyphenols and narirutin were significantly affected (*p* < 0.05) by different model terms. Particle size was affected by the quadratic protein term and the pumpkin oil–salt interaction term. PDI was influenced by the linear soy protein term and its quadratic term, as well as by the quadratic salt term. Furthermore, the ζ-potential was influenced by the linear pumpkin oil term, as well as by the soy protein–salt and salt–pumpkin oil interactions. Likewise, the retention of total polyphenols and narirutin was affected by the linear effect of soy protein.

Regarding particle size, the quadratic effect of soy protein shows a “saddle” response (Figure 1a,b), indicating the presence of an optimal level of this component in the system. The results suggest that extreme levels of soy protein in the formulation reduce droplet diameter, while intermediate levels increase it. This behavior may be because, at low concentrations, the protein readily adsorbs at the oil–water interface, forming a stabilizing layer; whereas at higher concentrations, it can generate greater coverage at the interface or even contribute to steric effects that promote droplet size reduction. Conversely, intermediate concentrations may not be sufficient to fully stabilize the interface during homogenization, thereby causing the formation of larger-diameter droplets [38,39]. The smaller the diameter of the droplets, the more stable they remain and the lower the risk of flocculation and coalescence [40].

In this context, Awlqadr et al. [41] presented different results, studying the emulsifying capacity of chickpea hull protein (1%, 3%, and 5%) and soy protein in lutein-loaded oil-in-water nanoemulsions. They found that at intermediate levels of chickpea hull protein (3%), the particle size was smaller and more stable (244.38 nm). They also associate this with the adsorption of the proteins at the oil–water interface. This discrepancy could be related to the greater structural flexibility of the soy protein in our study, which favors its reorganization and adsorption at the interface under the evaluated conditions [42].

Furthermore, the interaction effect between pumpkin and salt (Figure 1c) explains why gradually decreasing the oil concentration, at low salt levels, leads to a reduction in droplet diameter. This behavior can be explained by the fact that a lower proportion of the oil phase facilitates its dispersion during the homogenization process, favoring the formation of smaller, more uniform droplets. Similarly, at low salt concentrations, the ionic strength of the medium is lower, allowing greater maintenance of electrostatic interactions between proteins adsorbed at the oil–water interface. The combination of these factors contributes to a more homogeneous droplet distribution in the nanoemulsion [43].

Regarding the PDI, Figure 1d shows the linear effect of soy protein: increasing its proportion decreases the PDI value up to a certain point, beyond which it tends to increase again, a behavior reflected in the quadratic effect of soy protein also observed. At intermediate soy protein levels (1.65), the PDI remained lower, suggesting stable and monodisperse populations [44]. At high protein levels, the PDI deviates from 0, indicating polydisperse populations. Conversely, the quadratic effect of salt showed a different trend, with lower PDI values obtained at extreme levels. This behavior could be related to changes in the ionic strength of the medium, which modify the interactions between adsorbed proteins at the interface. Furthermore, the presence of salt can influence the charge state of proteins, especially near their isoelectric points where electrostatic repulsions decrease. Taken together, these factors may influence how the proteins organize themselves at the oil–water interface and, therefore, affect the uniformity of droplet size in the nanoemulsion [45].

Similar results were presented in a study evaluating nanoemulsions stabilized with soy protein isolate and phosphatidylcholine [46]. They observed that the PDI decreased at intermediate protein levels; as protein content increased from 1.0 to 1.5%, the nanoemulsions became more monodisperse, but above 1.5–2%, the PDI tended to increase. They explain that this could be due to excess unabsorbed protein and flocculation caused by depletion. Furthermore, in diosgenin nanoemulsions stabilized with soy protein, a low PDI (~0.17) was associated with high stability; however, this stability was maintained only up to NaCl concentrations of approximately 0.3 M, beyond which increased ionic strength favored destabilization processes and led to broad droplet size distribution [47].

Regarding the ζ-potential, which reflects the surface charge of the particles and provides information about the repulsive forces between them, higher absolute ζ-potential values favor greater electrostatic repulsion between the particles, which helps to prevent aggregation phenomena and, therefore, improves the stability of the system [48,49].

Increasing the oil content had a positive linear effect on the ζ-potential, meaning that although the ζ-potential values obtained were negative, they tended to approach zero as the oil content increased. This may be due to changes in interfacial organization and protein adsorption at the oil–water interface, where ionizable protein groups generate a negative charge on the droplet surface [50,51]. With higher oil content, droplet size increases, and there is also greater adsorption of proteins at the interface; however, the absolute value of the ζ-potential decreases, reflecting less electrostatic repulsion between the droplets [50]. Preferential adsorption and displacement depend on the composition and nature of the proteins [52]. This indicates that the ζ-potential is strongly dependent on oil concentration and on the dynamic behavior of proteins at the interface, which are key factors in controlling the stability of protein nanoemulsions [50,53].

As regards the pumpkin oil–salt interaction effect, it suggests that, at low salt levels, the gradual decrease in the oil proportion was associated with an increase in the absolute value of the ζ-potential, indicating greater system stability. This behavior is consistent with the results on particle size, since higher ζ-potential values favor greater electrostatic repulsion between droplets, reducing the probability of aggregation and contributing to the formation of smaller droplet systems.

In this way, Sun et al. [54] obtained similar results when preparing linseed oil nanoemulsions from homologous linseed gum. They evaluated the stability of the nanoemulsions as a function of storage temperature, pH, and sodium chloride concentration (100–500 mM/L, pH 5.8, 4 °C). They found that the absolute value of nanoemulsion potential decreased with increasing sodium chloride concentration, attributing this to the fact that when salt was added, Na^+^ ions were adsorbed onto the electronegative polysaccharide, reducing the ζ-potential and, consequently, promoting droplet aggregation.

Furthermore, the soy protein–pumpkin oil interaction effect showed that, at a high protein level, the gradual decrease in pumpkin oil also increased the absolute value of the ζ-potential. This result could be due to greater protein availability in the system, which favors adsorption at the oil–water interface, thereby increasing the droplets’ surface charge and, consequently, the electrostatic repulsion between them. Taken together, these results could indicate that both the soy protein–pumpkin oil ratio and the salt proportion influence the droplet surface charge and, therefore, the electrostatic stability of nanoemulsion [55].

For total polyphenols and narirutin, a linear effect of soy protein was observed, suggesting that, at intermediate oil and salt levels, increasing the protein ratio enhanced the retention of both compounds. This effect may be related to the ability of soy proteins to interact with phenolic compounds through different types of molecular interactions. Hydrogen bonding may play an important role, as narirutin contains several hydroxyl groups that can interact with polar functional groups in proteins. In addition, hydrophobic interactions may occur at the oil–water interface, where partial protein unfolding can expose nonpolar regions capable of interacting with the aromatic moieties of polyphenolic compounds. Although salt is not expected to form direct interactions with these compounds, it can modify the physicochemical environment of the system and potentially affect protein conformation, thereby influencing protein–polyphenol interactions [54,56]. These interactions may contribute to the retention of polyphenolic compounds within the nanoemulsion system and consequently reduce their exposure to degradation.

Despite this observed effect, the response-surface model for narirutin retention exhibited the lowest coefficient of determination among the models evaluated (R^2^ = 0.5005), indicating a relatively limited fitting capacity. Thus, approximately 50% of the variability in narirutin retention was explained by the formulation variables included in the experimental design, whereas the remaining variability may be associated with experimental variation and other factors not considered in the model. The lower fitting capacity may be related to the complexity of the mechanisms governing the retention of individual phenolic compounds, which may involve specific interactions with the different components of the nanoemulsion, as well as partitioning phenomena and differences in affinity for the oil, protein, and aqueous phases. Therefore, although the results indicate that soy protein plays a role in narirutin retention, the formulation variables investigated may not fully explain the variability observed for this compound. Consequently, predictions generated by the narirutin retention model should be interpreted with caution and restricted to the experimental domain investigated, particularly when quantitative prediction is required.

Similar results were reported by Sun et al. [57] in their study of the encapsulation of ferulic acid in quinoa protein nanoparticles and zein. They found that encapsulation efficiency increased with protein content (81.2% for quinoa albumin). They attributed this to the increased surface area, which provided the protein with intimate, robust contact with the phenolic compounds, enabling more efficient encapsulation.

### 3.2. Optimization and Validation

Optimization of the components used in the nanoemulsion formulation in this study was performed as previously described in the Materials and Methods section. The desirability function approach was used. In this study, the optimal nanoemulsion formulation was determined using Design-Expert software’s numerical optimization tool. Based on the maximum desirability of the multiple responses (0.935), the optimal formulation includes 3% soy protein, 4% pumpkin oil, and 0.34% salt.

Table 3 shows the predicted and experimental values obtained values for the optimal formulation, together with the corresponding prediction error.

The experimental values showed good agreement with the model predictions for total polyphenol retention and narirutin retention, with prediction errors of 9.07% and 6.43%, respectively. In contrast, higher deviations were observed for particle size (28.74%), PDI (15.54%), and ζ-potential (19.06%), indicating lower prediction accuracy for these physicochemical properties [58]. The greater deviation observed for particle size may be related to the relatively high experimental variability observed for this response among the replicated center points, as discussed above. Overall, the validation results indicate that the models provided a more accurate prediction of polyphenol retention than of the physicochemical characteristics of the nanoemulsion. Therefore, the optimized formulation should be considered an experimentally validated formulation within the investigated design space, while the differences between predicted and experimental values highlight the inherent variability associated with the preparation and characterization of the system. Regarding ζ-potential, the experimentally obtained value was −20.17 mV. This value is considerably below the ±30 mV magnitude commonly associated with systems stabilized predominantly by electrostatic repulsion, while remaining outside the ±5 mV range generally associated with very low surface charge [59]. Therefore, the ζ-potential suggests a moderate electrostatic contribution to the stability of the optimized formulation; however, other stabilization mechanisms, particularly those associated with the interfacial layer provided by soy protein, may also contribute to the overall stability of the emulsion.

### 3.3. Polyphenol Profile of Orange Peel Extract and Nanoemulsions Loaded with Orange Peel Extracts

The polyphenol profile of orange peel extracts (Table 4) was characterized by an abundant number of flavanones as the main compounds, and a high diversity of flavones and hydroxycinnamic acid derivatives. Hesperidin, narirutin, and apigenin 6,8-di-C-glucoside were the main compounds present in the extract, which represented 74% of the total amount of polyphenols; in total, twenty-four compounds were quantified in the obtained orange extract. The present results are in concordance with previous results [27,60]. The number of detected compounds in the optimal nanoemulsion was reduced to nineteen; only the majority hydroxycinnamic acids, ferulic acid glycoside, and feruloylcoumarate were detected. The distribution and amount were similar to those observed in orange extract. Flavanones were the subfamily of the orange extract that recovered more than 80% in the nanoemulsion, followed by flavones and phenolic acids, which were the least retained in the nanoemulsion, probably due to two main factors: the lower amount in the extract and their hydrophilic character (Table 4).

Individually, narirutin showed a high recovery rate, close to 96%, whereas naringenin triglycoside and diosmin also exhibited recoveries above 80%. A closer examination of the flavone profile revealed an interesting intra-family pattern. Major C-glucosides, such as apigenin and vitexin derivatives, exhibited high recovery rates of up to 75%, whereas luteolin 7-O-β-D-glucoside and the free flavones (1, 2, and 3) showed a drastic reduction, with recovery rates below 20%. This behavior may also be related to their low initial concentrations in the orange extract and their affinity for the aqueous phase, which could have limited their incorporation into fat globules. In this context, other authors reported an encapsulation efficiency of 74.3% for liposomes loaded with a flavonoid-rich fraction obtained from *Malpighia emarginata* pomace extract, in which flavonols were the predominant compounds [61]. Similarly, high polyphenol recoveries, ranging from 62.3% to 100%, have been reported for other delivery systems. However, in agreement with the findings of the present study, these authors also observed that phenolic compounds with a higher degree of polarity showed lower encapsulation efficiency values [62].

### 3.4. Physicochemical Properties of Films

The physicochemical properties of the developed control film, composed solely of chitosan and gelatin (CGF-C), a film containing orange peel extract (CGF-Ex), and a film containing nanoemulsions loaded with orange peel extract (CGF-N), are shown in Table 5. The results reveal significant modifications (*p* < 0.05) in the structural, hydration, and optical characteristics of the films as a consequence of the addition of orange peel extract or nanoemulsions loaded with orange peel extract.

Film thickness is an important parameter because it directly influences mechanical resistance, barrier performance, and mass transfer phenomena [63]. The control film (CGF-C) exhibited a thickness of 0.16 mm without statistical differences (*p* > 0.05) compared with the values obtained for CGF-Ex. These findings indicate that the direct incorporation of orange peel extract did not significantly alter the overall film-forming process or the distribution of polymeric chains within the matrix. These results agree with Li et al. [20], who reported that the addition of seaweed extracts into gelatin/zein films had no effect on film thickness. On the other hand, the incorporation of nanoemulsions loaded with orange peel extract resulted in a substantial increase (*p* < 0.05) in thickness, reaching 0.42 mm. This increase may be attributed to the incorporation of the nanoemulsion into the polymer network, which likely increased the overall solid content of the film and contributed to the observed increase in thickness [23]. However, since no microscopic characterization (e.g., SEM or AFM) was performed, the specific distribution of the nanoemulsion droplets within the polymer matrix cannot be confirmed. Nevertheless, this finding is consistent with previous studies. Almasi et al. [64] reported that biopolymer films containing essential oil-loaded microemulsions exhibited greater thickness than the corresponding control films. Similarly, Shiryanpour et al. [10] reported that chitosan films containing single and double nanoemulsions of *Oliveria decumbens* Vent. essential oil and eggplant anthocyanins exhibited greater thickness (0.156 and 0.179 mm, respectively) than the control films (0.137 mm).

The moisture content of edible films is a critical factor affecting their mechanical stability, flexibility, and susceptibility to microbial degradation [65]. The CGF-C film presented (Table 5) the lowest moisture content (*p* < 0.05), whilst the addition of orange peel extract or nanoemulsions loaded with orange peel extract significantly increased (*p* < 0.05) this parameter, with values of 18.07% and 11.96% in CGF-Ex and CGF-N, respectively. In the case of CGF-Ex, this behavior may be associated with the presence of hydrophilic phenolic compounds and other polar constituents present in the extract, which enhance water retention through hydrogen bonding interactions with chitosan and gelatin molecules, as reported by Tao et al. [3]. Thus, Kumar et al. [8] found that chitosan edible films enhanced with pomegranate peel extract had a higher moisture content than the control film. On the other hand, the incorporation of the extract through nanoemulsions resulted in moisture contents closer to, or lower than, those of the control film, in agreement with what was reported by Shiryanpour et al. [10]. According to Rui et al. [9], this reduction in moisture may be related to interactions between the nanoemulsion components and the chitosan matrix, which could modify the interactions between chitosan and water and consequently reduce the availability of hydrophilic groups for hydrogen-bond formation with water. However, this proposed mechanism should be interpreted cautiously, as no direct structural characterization was performed in the present study to confirm these intermolecular interactions.

Film solubility is another key parameter for edible packaging applications, as it reflects the material’s stability under humid conditions and determines its potential performance during food storage [63]. The CGF-C film had the highest solubility value (*p* < 0.05), followed by CGF-Ex and CGF-N, which showed the lowest value (*p* < 0.05). The results showed (Table 5) that the addition of orange peel extract significantly reduced film solubility, suggesting the formation of additional intermolecular interactions between the extract constituents and the polymer matrix. The addition of nanoemulsion loaded with orange peel extract significantly improved the water resistance of the films. The enhanced stability may be attributed to the presence of hydrophobic domains generated by the nanoemulsion droplets, which reduced water penetration and limited polymer chain relaxation [9]. The swelling ratio provides additional information regarding the interaction between the film matrix and water. The CGF-C film showed (Table 5) a significantly (*p* < 0.05) higher swelling ratio than CGF-Ex and CGF-N, indicating extensive water uptake and significant matrix expansion upon hydration. The reduction observed after the addition of orange peel extract or nanoemulsions loaded with orange peel extract suggests that the bioactive compounds promoted stronger intermolecular associations within the polymeric network, thereby limiting water diffusion and reducing structural expansion.

Water vapor permeability (WVP) is one of the most important properties of packaging materials because it determines the ability of the film to act as a moisture barrier [11]. The WVP values of CGF-C, CGF-Ex and CGF-N ranged from 7.91 × 10^−9^ to 9.24 × 10^−9^ g/m s Pa, with no significant differences (*p* > 0.05) among the formulations (Table 5). These results indicate that neither the direct addition of orange peel extract nor its incorporation through nanoemulsions significantly affected water vapor transport through the films. The values obtained in the present study are consistent with those reported by Tao et al. [3], who observed no significant differences in WVP between control films and films incorporated with *Galla chinensis* extract. However, other studies have reported contrasting results. For instance, Chen et al. [12] found that incorporating loquat peel extract into chitosan-based films reduced WVP by 44.5% compared with the control treatment. Similarly, Cai et al. [4] reported that guar gum–chitosan films containing orange essential oil nanoemulsions exhibited a 25.38% decrease in WVP relative to the control films. Water vapor permeability is strongly influenced by the solubility and diffusion of water molecules within the biopolymer matrix [66]. Therefore, the discrepancies observed among studies may be attributed to differences in the chemical composition of the incorporated bioactive compounds, their interactions with the polymer network, and the resulting modifications in film microstructure and water transport properties.

The optical properties of edible films are particularly relevant because they influence consumer acceptance and the ability of the packaging material to protect food products from light-induced deterioration [65,67]. The addition of orange peel extract or nanoemulsions loaded with orange peel extract provoked significant differences in the color parameters among the analyzed films (Table 5). Regarding lightness (L*), the CGF-C film exhibited the highest value (*p* < 0.05), reflecting its brighter and more transparent appearance. The incorporation of orange peel extract significantly decreased the L* value to 70.03, whereas films containing nanoemulsions loaded with orange peel extract showed a further reduction in lightness, exhibiting the lowest L* values among all treatments (*p* < 0.05). This effect may be attributed to the enhanced dispersion of color-contributing compounds within the film matrix promoted by peel extracts, resulting in a darker and less transparent film appearance. These results agree with those reported by Chen et al. [12], who found a reduction in the lightness of edible chitosan films incorporated with loquat peel extract as the concentration increased. As regards the redness (a*) coordinate, CGF-C films showed the highest value (*p* < 0.05), while the CGF-Ex had the lowest value without statistical differences with CGF-N. More pronounced differences were observed for the yellowness (b*) coordinate. Again, CGF-C had the lowest value (*p* < 0.05), whilst the addition of orange peel extract or nanoemulsions loaded with orange peel extracts significantly increased b* values, with CGF-Ex being the highest value (*p* < 0.05). This increase in the b* coordinate may be attributed to the presence of flavonoids and other naturally occurring yellow-orange pigments in the extract. These compounds enhance the yellow color intensity of the film matrix, leading to higher b* values, as reported by Terzioğlu et al. [19] and Umaña et al. [68].

Opacity measurements further support the observed changes in optical behavior. While CGF-C and CGF-Ex exhibited comparable (*p* > 0.05) opacity values of 27.39% and 25.15%, respectively, the CGF-N films showed an increase (*p* < 0.05), achieving values of 49.15% (Table 5). This enhanced opacity could be likely associated with increased light scattering caused by the presence of numerous nano-sized droplets distributed throughout the matrix [69].

**Figure 2:**
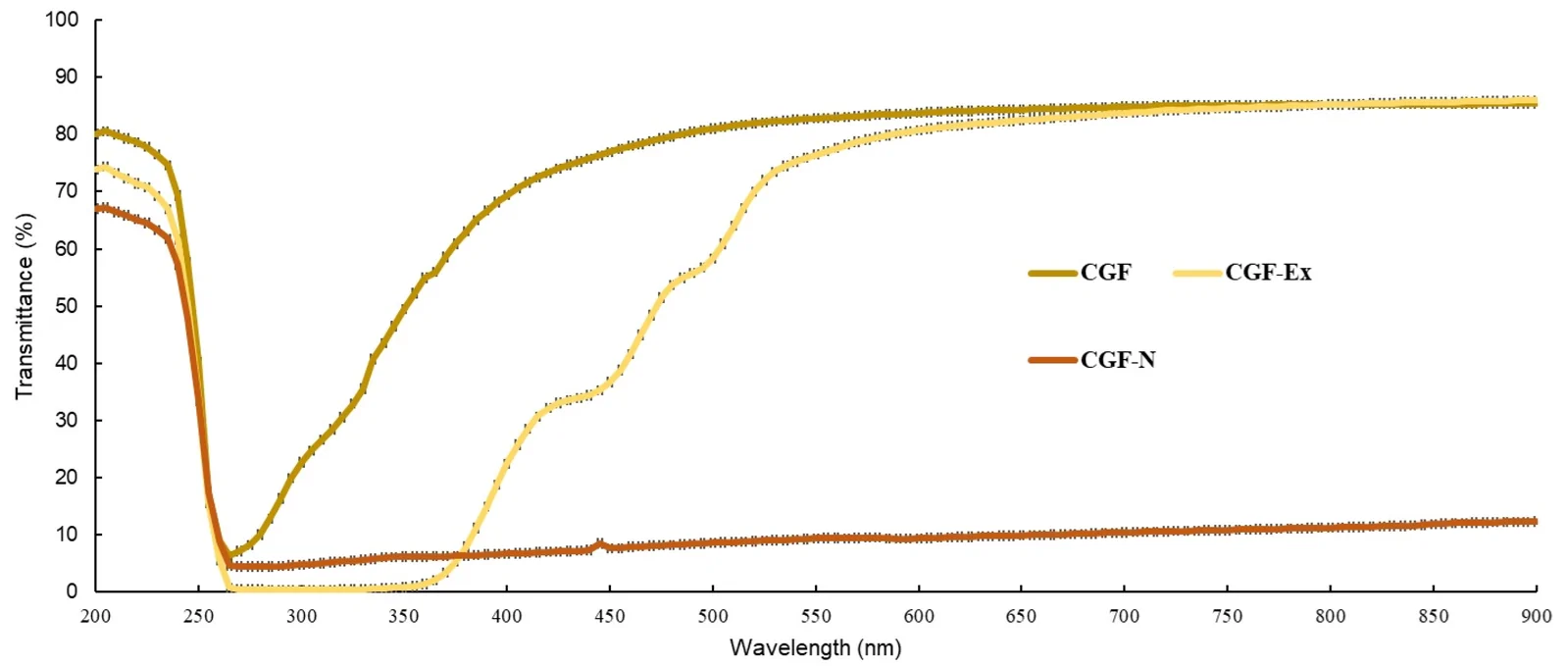
The optical transmittance values obtained from chitosan/gelatin edible films (CGF-C); chitosan/gelatin edible film added with orange peel extract (CGF-Ex) or chitosan/gelatin edible film added with nanoemulsions loaded with orange peel extract (CGF-N).

The optical transmittance values obtained from analyzed films (Figure 2) showed marked differences as a function of formulation, highlighting the influence of extract or nanoemulsion addition on light transmission properties. Thus, CGF-C had the highest overall transparency across the evaluated spectral range (200–900 nm), with transmittance increasing progressively from the UV to the visible and near-infrared regions. This behavior is characteristic of homogeneous polysaccharide–protein matrices and indicates a relatively low capacity to attenuate incident radiation [70]. The incorporation of orange peel extract (CGF-Ex) significantly reduced film transmittance, particularly within the ultraviolet and lower visible regions. A pronounced decrease was observed between approximately 250 and 500 nm. This fact might be due to phenolic compounds, flavonoids, and other chromophoric constituents present in the extract absorbing and scattering incident light. These results agree with those reported by Tone et al. [71], who mentioned a similar behavior of polypropylene and polylactic acid films added with orange peel extracts.

CGF-N displayed the lowest transmittance values, indicating a substantial enhancement in opacity and light-barrier performance. The marked reduction in light transmission can be attributed (*i*) to the presence of bioactive compounds from the orange peel extract and (*ii*) to increased light scattering generated by the nanoscale dispersed phase, as mentioned by Shiryanpour et al. [10].

### 3.5. Polyphenol Profile of Chitosan/Gelatin Films and Release

As expected, the control films (without orange extract) did not present polyphenols, whereas they were detected in both the free-extract and nanoemulsion-loaded films. Concerning CGF-Ex and CGF-N, their polyphenolic profiles were identical, consisting of thirteen compounds (Table 6). This indicates that while the original orange peel extract contained approximately 24 compounds, the minority compounds were not detected once incorporated into the films.

Similar to the trend observed in the raw orange peel extract and nanoemulsion loaded with orange peel extracts, hesperidin and narirutin remained the predominant compounds in both CGF-Ex and CGF-N. While the total polyphenol concentration and the three studied families were significantly higher in CGF-Ex than in CGF-N (*p* < 0.05), a different trend emerged when analyzing the recovery rate. Based on the actual initial concentration loaded into the films (Supplementary Table S2), the nanoemulsion successfully preserved polyphenol stability compared to the free extract across all studied phenolic compounds. Consequently, the total polyphenol and subfamily recovery rates were significantly higher in CGF-N than in CGF-Ex (*p* < 0.05). Specifically, the recovery of total polyphenols and total flavanones reached up to 80% in CGF-N, whereas it only reached 66.8% and 75.9% in CGF-Ex, respectively. These findings highlight that incorporating polyphenol-rich extracts into chitosan-gelatin films via nanoemulsion effectively protects polyphenols from degradation and loss.

The kinetics of polyphenol release from the matrix are related to their bioactivity. Higher binding to the matrix limits polyphenol release, whereas low binding can lead to degradation. Therefore, controlling release is a key factor in developing bioactive films. The proposed film is designed to be used in fresh meat products (e.g., burgers) or cooked products such as ham. Thus, a food simulant solution representative of meat products was employed for release studies, and the temperature was set at 4 °C to replicate the storage conditions of these products.

**Figure 3:**
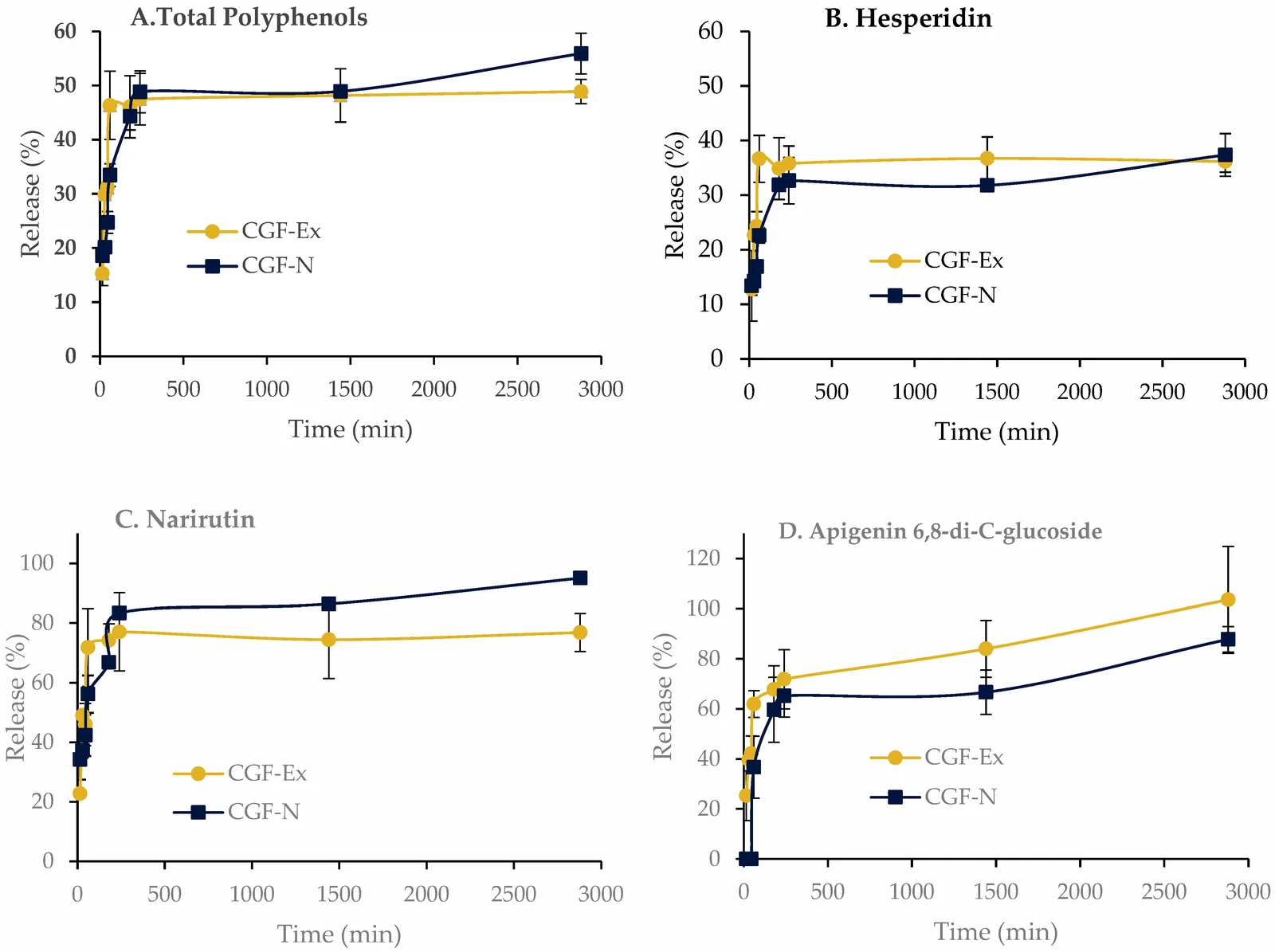
Percentage of (**A**) total polyphenols, (**B**) hesperidin, (**C**) narirutin, and (**D**) apigenin 6,8-di-C-glucoside released at 4 °C into a simulated food solution (10% *v*/*v* aqueous ethanol) from chitosan/gelatin film added with orange peel extract (CGF-Ex) and chitosan/gelatin film added with orange peel extract loaded.

The total polyphenol release of both CGF-Ex and CGF-N films was lower than 60% (Figure 3), which may be related to possible hydrogen bonding and ionic interactions between the amine/hydroxyl groups of chitosan and the phenolic groups. Similar results were reported by Thi Nguyen et al. [35]. However, the release behavior was intrinsic to each compound. For example, while hesperitin release reached only around 40% in both films, narirutin release reached approximately 75% and 84% for CGF-Ex and CGF-N, respectively.

The kinetic parameters of the Peleg model (Table 7) for the release of individual and total (poly)phenolic compounds from CGF-Ex and CGF-N samples revealed that the release of total polyphenols and flavanones was faster in CGF-Ex than in CGF-N. However, the release kinetics were statistically equal in the case of apigenin (*p* > 0.05). Interestingly, the highest infinite release concentration at equilibrium was reported for CGF-N, indicating that encapsulation successfully prevented active compound degradation. This controlled release behavior was further confirmed by the Weibull model (Supplementary Table S3), where the release time constant (τ), which represents the time required to reach 63.2% of total release, was estimated at 35.7 min for CGF-Ex, while for CGF-N it more than doubled to 83.2 min. Similar significant differences were also observed for total and individual flavanones (*p* < 0.05). The only exception was apigenin 6,8-di-C-glucoside, which presented the highest τ with no significant differences between the two films. It should be noted that the release behavior observed in the present study was evaluated under controlled food-simulant conditions and, therefore, should be interpreted within the specific experimental conditions employed. Although the use of food simulants provides a standardized approach for characterizing the release profiles and comparing the behavior of CGF-Ex and CGF-N films, the migration and release of active compounds may be affected by factors associated with the packaged food and storage environment, including food composition, polarity, pH, water activity, temperature, and storage time. Consequently, the release kinetics and mechanisms identified in this study cannot be directly extrapolated to all food matrices or storage conditions. Further studies under different storage temperatures and using representative food matrices are required to validate the observed release behavior and to determine the performance of these films under realistic food-packaging conditions. Authors such as Liu et al. [72] also reported that tea polyphenols incorporated into gelatin films exhibit a slower and more controlled release when they were encapsulated compared to when they were added as a free extract.

Concerning the mass-transfer behavior in the food simulant, distinct release profiles were observed between the two films (Supplementary Table S3). CGF-Ex exhibited a sigmoidal release profile, as indicated by β > 1, whereas CGF-N showed β < 1, consistent with a release profile characterized by a decreasing release rate and the absence of the pronounced lag phase associated with sigmoidal behavior [73,74]. Therefore, the β parameter was interpreted primarily as a descriptor of the shape of the release profile rather than as direct evidence of a specific molecular release mechanism. The sigmoidal behavior observed for CGF-Ex may be associated with an initial period during which polymer swelling, matrix relaxation, and interactions between the polyphenolic compounds and the chitosan/gelatin network could delay their diffusion into the food simulant. However, these mechanisms were not directly characterized in the present study and should therefore be considered possible contributing factors. In contrast, the different release profile observed for CGF-N may be related to the incorporation of the extract within the nanoemulsion structure, which can modify the partitioning and diffusion of the polyphenolic compounds through the film matrix. The significantly higher τ value observed for CGF-N further indicates a slower release process and a more sustained release profile. This behavior may be associated with the additional mass-transfer resistance introduced by the nanoemulsion structure; however, direct evidence of this mechanism would require further structural and transport characterization. Similar non-immediate release behavior has been reported for polyphenols incorporated into chitosan-based edible films, with Ribeiro et al. [75] reporting β values between 1.48 and 1.66.

### 3.6. Antioxidant Activity

The antioxidant capacity of the developed chitosan/gelatin-based films was evaluated using two complementary analytical approaches with different mechanisms of action, namely ferric reducing antioxidant power (FRAP) assay and the 2,2-diphenyl-1-picrylhydrazyl (DPPH) radical scavenging method. These assays provide valuable information regarding the reducing potential and free radical neutralization ability of the films, respectively. The results obtained (Table 5) showed that the incorporation of orange peel-derived bioactive compounds (direct addition or as nanoemulsion) significantly enhanced the antioxidant properties of the reformulated films (*p* < 0.05) compared with the control formulation.

Regarding the FRAP assay, the control film exhibited the lowest (*p* < 0.05) antioxidant activity, with a value of 103.08 µg Trolox equivalent (TE) per g of film. The incorporation of orange peel extract into the chitosan/gelatin matrix resulted in a remarkable increase in antioxidant activity, reaching 722.07 µg TE per g of film. This represented an approximately seven-fold increase compared with CGF-C. Similarly, the film loaded with orange peel extract in nanoemulsions also exhibited a significantly higher (*p* < 0.05) FRAP value (607.08 µg TE per g of film) than the control. However, this value was lower (*p* < 0.05) than that obtained for the film incorporating the non-encapsulated extract. In this way, Castro-Vazquez et al. [76] reported that the nanoemulsified encapsulation process had no effect on the antioxidant capacity of orange peel extract measured with FRAP assays. Then, this reduction in antioxidant effect shown by CGF-N may be attributed to interactions between phenolic compounds and the chitosan–gelatin network components that might reduce the availability of active hydroxyl groups responsible for the antioxidant effect, limiting their antioxidant effectiveness, as mentioned by Rodrigues et al. [15].

The DPPH assay showed a similar trend. Both films enriched with orange peel extract and orange peel extract loaded into nanoemulsions exhibited significantly greater radical scavenging activity than the control film (*p* < 0.05). The films containing orange peel extract had a DPPH value of 118.05 µg TE/g films, while the films added with orange peel extract loaded into nanoemulsions exhibited a slightly higher value of 134.70 µg TE/g films without differences between them (*p* > 0.05). These results indicated that the antioxidant compounds retained their ability to neutralize free radicals regardless of whether they were incorporated as free extract or encapsulated within a nanoemulsion system.

Orange peel is recognized as a source of bioactive compounds, mainly phenolic acids and flavonoids with a huge antioxidant activity [77]. In addition, this extract has been used for the functionalization of edible films, improving their antioxidant properties. In this sense, Umaña et al. [68] reported that edible films elaborated with pectin and added with orange residue extracts showed values for antioxidant capacity of 18.00 and 12.00 mg TE/g, measured with ABTS and FRAP assays respectively. More recently, Gouda et al. [78] found that biopolymer composite films elaborated with arabic gum and carboxymethyl cellulose and loaded with orange peel extract showed an antioxidant capacity measured with the FRAP assay of 648 μM Trolox/mg extract.

### 3.7. Antibacterial Properties

The antibacterial activity of the developed CGF-C, CGF-Ex and CGF-N films was assessed against four bacterial strains, namely *Serratia marcescens, Escherichia coli, Bacillus* sp., and *Listeria innocua*. None of the tested formulations exhibited any antibacterial activity against the strains evaluated under the experimental conditions employed. Thus, both the control film and the films containing orange peel extract, either in free form or incorporated in a nanoemulsion, might be classified as inactive under the experimental conditions employed. These results suggest that the concentration of bioactive compounds released from the film matrix was insufficient to inhibit bacterial growth, despite the well-documented antimicrobial properties of chitosan and orange-peel extract reported in the literature [79,80]. Furthermore, interactions between the polymeric network and the incorporated extract may have limited the diffusion and bioavailability of bioactive compounds in the agar medium. Therefore, direct-contact assays under food-relevant conditions would provide complementary information and should be considered in future studies to further characterize the antimicrobial effectiveness of the developed films. The absence of antibacterial effects highlights the importance of optimizing both the concentration and release kinetics of active compounds to enhance the antimicrobial performance of biodegradable edible films intended for food packaging applications.

## 4. Conclusions

This study demonstrated the feasibility of using orange peel extract (OPE)-loaded nanoemulsions as a delivery strategy for the development of chitosan/gelatin-based active films. Nanoencapsulation provided greater protection of OPE polyphenols during film processing and enhanced their preservation and antioxidant functionality compared with the incorporation of the free extract. The incorporation of nanoemulsions also improved relevant film characteristics, particularly light-barrier performance and resistance to water interaction, while enabling a more gradual release of polyphenolic compounds. These findings highlight the potential of nanoemulsion technology to improve the stability and functional performance of natural extracts incorporated into biodegradable packaging matrices.

The main contribution of this work is the combination of citrus by-product valorization with nanoemulsion-based delivery in a chitosan/gelatin film, providing a promising approach for the development of bioactive packaging materials with enhanced protection and controlled-release properties. However, the absence of antibacterial activity under the conditions tested and the evaluation of release behavior exclusively using food simulants represent relevant limitations. Further research should assess the performance of these films with real food matrices and under different storage temperatures and conditions, as well as evaluate their effectiveness during actual food storage. Such studies are necessary to confirm their practical applicability and potential contribution to more sustainable food-packaging systems.

## Figures and Tables

**Table 1:** Experimental and coded treatment combinations used in the Box–Behnken design, along with the measured response variables of the nanoemulsions.

	Experimental Values			Response Variables				
Run	X_1_ Soy Protein (%)	X_2_ Pumpkin Oil (%)	X_3_ Salt (%)	Y_1_ Particle size (nm)	Y_2_ PDI	Y_3_ ζ-Potential (mV)	Y_4_ TP Retention (%)	Y_5_ Narirutin Retention (%)
1	1.65 (0)	10.50 (0)	0.30 (0)	1001.80	0.590	−21.33	82.73	85.41
2	1.65 (0)	10.50 (0)	0.30 (0)	651.85	0.446	−19.76	77.12	78.39
3	1.65 (0)	10.50 (0)	0.30 (0)	1582.66	0.414	−18.90	37.71	35.59
4	1.65 (0)	17.00 (+1)	0.60 (+1)	1681.66	0.323	−14.06	49.53	47.54
5	1.65 (0)	4.00 (−1)	0.60 (+1)	268.26	0.454	−20.16	56.93	55.05
6	1.65 (0)	17.00 (+1)	0.00 (−1)	256.63	0.373	−1.23	80.31	81.62
7	1.65 (0)	4.00 (−1)	0.00 (−1)	1944.70	0.222	−30.30	75.34	78.29
8	3.00 (+1)	10.50 (0)	0.60 (+1)	289.50	0.318	−5.24	75.96	80.69
9	0.30 (−1)	10.50 (0)	0.60 (+1)	191.33	0.753	−17.80	76.78	88.46
10	3.00 (+1)	10.50 (0)	0.00 (−1)	135.73	0.241	−9.49	77.98	84.02
11	0.30 (−1)	10.50 (0)	0.00 (−1)	198.23	1.000	−18.96	84.66	88.17
12	3.00 (+1)	17.00 (+1)	0.30 (0)	220.73	0.519	−4.89	87.66	93.87
13	0.30 (−1)	17.00 (+1)	0.30 (0)	191.33	0.959	−17.95	83.48	87.63
14	3.00 (+1)	4.00 (−1)	0.30 (0)	157.26	0.252	−26.40	87.60	94.11
15	0.30 (−1)	4.00 (−1)	0.30 (0)	102.36	1.000	−17.56	46.99	42.68

PDI: Polydispersity index; TP: Total polyphenols; Treatments 1–3 correspond to the replicated center points, while treatments 4–15 represent the Box–Behnken design combinations.

**Table 2:** Analysis of variance and estimated regression coefficients for the regression models of the response variables in the measured nanoemulsions.

Response	Means + SE	Model	R^2^	Lack of Fit	Anova *p*-Value	Polynomial Equation
Particle size (nm)	1078.77 ± 179.82	Quadratic	0.9154	0.9737	0.0313	1078.77 + 15A−15.28B − 13.07C − 6.37AB + 40.17AC + 775.37BC *−872.48 A2 *−38.37B2 − 2.59C2
PDI	0.4832 ± 0.05	Quadratic	0.9643	0.5996	0.0041	0.4832 + 0.2977A * + 0.0307B + 0.0014C + 0.0769AB + 0.0811AC − 0.0706BC + 0.2169A^2^ * − 0.0176B^2^ − 0.1224C ^2^*
ζ-potential	−20 ± 2.24	Quadratic	0.9162	0.0598	0.0306	−20.00 + 3.28A + 7.04B * + 0.338C + 5.47AB * + 0.769AC − 5.74BC * + 3.43A^2^ − 0.134B^2^ + 3.69C^2^
TP retention (%)	70.24 ± 2.81	2Fl	0.7645	0.7594	0.0304	70.24 + 15.80A * + 4.97B + 6.39C−9.11AB−1.59AC−7.25BC
Narirutin retention (%)	73.74 ± 3.93	Linear	0.5005	0.6552	0.0470	73.74 + 16.31A * + 1.19B + 7.06C

SE: Standard error; PDI: Polydispersity index; TP: Total polyphenols; Notes: 0 < R^2^ < 1, close to 1 means more significant. A: soy protein. B: pumpkin oil. C: salt. * *p* < 0.05. Abbreviation: SE, standard error. PDI: polydispersity index.

**Table 3:** Predicted values vs. experimental values.

	Predicted	Experimental	Prediction Error (%)
Particle size (nm)	102.37	131.79	28.74
PDI	0.296	0.25	15.54
ζ-potential	−24.92	−20.17	19.06
Total polyphenol retention (%)	91.83	83.50	9.07
Narirutin retention (%)	89.83	95.61	6.43

PDI: polydispersity index.

**Table 4:** Polyphenolic profile of orange peel extract, nanoemulsions loaded with orange peel extract and recovery efficiency.

Family	Compound	Orange Peel Extract	Nanoemulsions Loaded With Orange Peel Extract	Recovery (%)
Phenolic acids	Ferulic acid glycoside	369.96± 5.40	15.77 ± 0.80	42.62 ± 2.66
	Feruloylcoumarate	86.29 ± 3.61	6.00 ± 0.10	69.52 ± 1.41
	Ferulate	25.69 ± 0.23	nd	-
	Ferulate	71.25 ± 3.20	nd	-
	Ferulate	71.01 ± 2.60	nd	-
	*p*-coumarate	32.09 ± 4.08	nd	-
	*p*-coumarate	32.68 ± 0.44	nd	-
Flavanones	Naringenin triglycoside	479.90 ± 70.37	38.95 ± 1.82	81.17 ± 4.64
	Hesperitin triglycoside	243.55 ± 16.44	17.36 ± 1.79	71.29 ± 9.01
	Eriocitrin *	128.21 ± 7.51	7.04 ± 0.51	54.90 ± 4.89
	Narirutin *	1857.12 ± 13.60	177.83 ± 5.60	95.61 ± 3.68
	Hesperidin *	4620.09 ± 133.35	362.65 ± 14.18	78.49 ± 3.76
	Dydimin *	355.86 ± 16.38	28.00 ± 2.34	78.68 ± 8.06
Flavones	Apigenin 6,8-di-C-glucoside	539.73 ± 27.53	37.53 ± 1.31	69.53 ± 2.96
	Diosmetin 6,8-di-C-glucoside	84.97 ± 11.26	4.60 ± 0.25	54.16 ± 3.58
	Diosmin *	57.06 ± 1.43	4.96 ± 0.30	86.84 ± 6.35
	Diosmetin glycoside	109.47 ± 1.89	7.46 ± 0.22	68.13 ± 2.49
	Luteolin 7-O-β-D-glucoside *	36.20 ± 4.93	0.37 ± 0.03	10.35 ± 0.97
	Isoorientin *	19.29 ± 0.70	0.38 ± 0.01	19.84 ± 0.60
	Vitexin-2-O-rhamnose *	107.02 ± 19.42	8.12 ± 0.27	75.90 ± 3.08
	Vitexin *	65.20 ± 1.53	3.29 ± 0.10	50.53 ± 1.81
	Flavone 1	24.82 ± 1.37	0.32 ± 0.02	13.08 ± 0.91
	Flavone 2	43.06 ± 1.07	0.56 ± 0.01	12.98 ± 0.39
	Flavone 3	39.88 ± 1.85	0.48 ± 0.01	11.92 ± 0.46
	Total Phenolic acids	683.98 ± 26.78	22.38 ± 0.15	32.72 ± 0.26
	Total Flavanones	7684.74 ± 202.18	631.84 ± 14.07	82.22 ± 2.24
	Total Flavones	1126.71 ± 32.98	68.08 ± 2.35	60.42 ± 2.55
	Total (poly)phenols	9495.42 ± 198.85	722.30 ± 13.66	76.07 ± 1.76

* Compounds confirmed by standard. nd: not detected. Values expressed as mg/mL.

**Table 5:** Physicochemical and antioxidant properties of chitosan/gelatin edible films (CGF-C); chitosan/gelatin edible film added with orange peel extract (CGF-Ex) or chitosan/gelatin edible film added with nanoemulsions loaded with orange peel extract (CGF-N).

	CGF-C	CGF-Ex	CGF-N
Thickness (mm)	0.16 ± 0.02 ^b^	0.16 ± 0.03 ^b^	0.42 ± 0.09 ^a^
Moisture (%)	10.08 ± 0.45 ^c^	18.07 ± 0.81 ^a^	11.96 ± 0.35 ^b^
Solubility (%)	64.73 ± 1.51 ^a^	53.35 ± 2.68 ^b^	31.86 ± 5.93 ^c^
Swelling ratio	12.93 ± 1.74 ^a^	2.68 ± 0.09 ^c^	5.44 ± 0.59 ^b^
WVP (g m^−1^ s^−1^ Pa^−1^) × 10^−9^	8.23 ± 1.28 ^a^	9.24 ± 0.21 ^a^	7.91 ± 1.48 ^a^
L*	74.48 ± 0.25 ^a^	70.03 ± 0.39 ^b^	62.95 ± 3.53 ^c^
a*	−1.13 ± 0.05 ^a^	−2.01 ± 0.35 ^b^	−1.45 ± 0.24 ^b^
b*	5.84 ± 0.46 ^c^	34.75 ± 1.89 ^a^	30.49 ± 2.86 ^b^
Opacity (%)	27.39 ± 4.71 ^b^	25.15 ± 4.89 ^b^	49.15 ± 7.79 ^a^
FRAP assay (µg TE/g film)	103.08 ± 8.61 ^c^	722.07 ± 52.82 ^a^	607.08 ± 49.72 ^b^
DPPH assay (µg TE/g film)	0.00 ± 0.00	118.05 ± 13.03 ^a^	134.7 ± 3.48 ^b^

TE: Trolox equivalents. WVP: water vapor permeability. Values followed by the same letter, in the same row, are not significantly different (*p* > 0.05) according to Tukey’s Multiple Range Test.

**Table 6:** Initial concentration, total released amount, and percentage retained of individual and total phenolic compounds from chitosan/gelatin edible film added with orange peel extract (CGF-Ex) and chitosan/gelatin edible film added with nanoemulsions loaded with orange peel extract (CGF-N).

Family	Compound	Concentration		Release		Retained in the Film	
		CGF-Ex	CGF-N	CGF-Ex	CGF-N	CGF-Ex	CGF-N
**Phenolic** **acids**	Ferulic acid glycoside	0.17 ± 0.01 ^a^	0.14 ± 0.01 ^b^	nd	nd	-	-
	Feruloylcoumarate	0.07 ± 0.00 ^a^	0.02 ± 0.00 ^b^	nd	nd	-	-
**Flavanones**	Naringenin triglycoside	0.98 ± 0.09 ^a^	0.68 ± 0.02 ^b^	0.86 ± 0.02 ^a^	0.70 ± 0.01 ^b^	12.21 ± 2.29 ^a^	0.00 ± 0.00 ^b^
	Hesperitin triglycoside	0.40 ± 0.05 ^b^	0.62 ± 0.04 ^a^	0.34 ± 0.01 ^b^	0.69 ± 0.06 ^a^	15.80 ± 1.65 ^a^	0.00 ± 0.00 ^b^
	Narirutin	3.55 ± 0.49 ^a^	2.73 ± 0.04 ^b^	2.73 ± 0.01 ^a^	2.60 ± 0.14 ^a^	23.15 ± 0.37 ^a^	4.97 ± 6.39 ^b^
	Hesperidin	12.41 ± 0.87 ^a^	8.83 ± 0.28 ^b^	4.49 ± 0.40 ^a^	3.30 ± 0.14 ^b^	63.84 ± 3.91 ^a^	62.64 ± 1.93 ^a^
	Dydimin	0.88 ± 0.02 ^a^	0.54 ± 0.03 ^b^	0.37 ± 0.03 ^a^	0.29 ± 0.01 ^b^	57.94 ± 4.27 ^a^	45.55 ± 2.46 ^b^
**Flavones**	Apigenin 6,8-di-C-glucoside	0.83 ± 0.10 ^a^	0.53 ± 0.02 ^b^	0.86 ± 0.14 ^a^	0.47 ± 0.02 ^b^	0.00 ± 0.00 ^b^	12.16 ± 5.09 ^a^
	Diosmetin 6,8-di-C-glucoside	0.11 ± 0.01 ^a^	0.06 ± 0.01 ^b^	nd	nd	-	-
	Diosmin	0.11 ± 0.01 ^a^	0.09 ± 0.01 ^b^	nd	nd	-	-
	Diosmetin glycoside	0.20 ± 0.00 ^a^	0.14 ± 0.00 ^b^	nd	nd	-	-
	Vitexin-2-O-rhamnose	0.13 ± 0.00 ^a^	0.11 ± 0.00 ^b^	nd	nd	-	-
	Vitexin	0.06 ± 0.00 ^a^	0.05 ± 0.00 ^a^	nd	nd	-	-
	Total (poly)phenols	19.89 ± 0.19 ^a^	14.55 ± 0.27 ^b^	9.64 ± 0.60 ^a^	8.05 ± 0.26 ^b^	51.33 ± 3.72 ^a^	44.37 ± 2.23 ^b^
	Total Phenolic acids	0.24 ± 0.01 ^a^	0.15 ± 0.01 ^b^	nd	nd	-	-
	Total Flavanones	18.22 ± 0.26 ^a^	13.41 ± 0.32 ^b^	8.78 ± 0.46 ^a^	7.59 ± 0.24 ^b^	51.81 ± 3.09 ^a^	43.43 ± 2.23 ^b^
	Total Flavones	1.43 ± 0.11 ^a^	0.99 ± 0.04 ^b^	0.86 ± 0.14 ^a^	0.47 ± 0.02 ^b^	40.12 ± 12.32 ^b^	52.48 ± 2.75 ^a^

Values are expressed in mg/g film as mean ± SD. nd: not detected. Different letters in the same row and for each studied parameter indicate significant differences according to Tukey’s test (*p* < 0.05).

**Table 7:** Peleg model kinetic parameters for the release of individual and total (poly)phenolic compounds from chitosan/gelatin edible film added with orange peel extract (CGF-Ex) or chitosan/gelatin edible film added with nanoemulsions loaded with orange peel extract (CGF-N), including the initial release resistance (k_1_), the constant related to the equilibrium release (k_2_), the estimated maximum release (M∞ = 1/k_2_), and the coefficient of determination (R^2^).

	CGF-Ex				CGF-N			
	k_1_ (h/%)	k_2_ (%^−1^)	M∞ (%)	R^2^	k_1_ (h/%)	k_2_ (%^−1^)	M∞ (%)	R^2^
Naringenin triglycoside	0.094 ± 0.010 ^b^	0.011 ± 0.000 ^a^	88.53 ± 2.05 ^b^	1.000 ± 0.000	0.20 ± 0.03 ^a^	0.010 ± 0.000 ^b^	103.49 ± 2.51 ^a^	0.999 ± 0.000
Hesperitin tryglicoside	0.062 ± 0.018 ^b^	0.012 ± 0.000 ^a^	84.30 ± 1.80 ^b^	1.000 ± 0.000	0.49 ± 0.10 ^a^	0.009 ± 0.001 ^b^	112.47 ± 15.54 ^a^	0.996 ±0.006
Narirutin	0.249 ± 0.022 ^b^	0.013 ± 0.000 ^a^	76.93 ± 0.59 ^b^	1.000 ± 0.000	0.61 ± 0.20 ^a^	0.011 ± 0.001 ^b^	95.41 ± 8.03 ^a^	0.996 ±0.005
Hesperidin	0.671 ± 0.240 ^b^	0.026 ± 0.000 ^a^	38.51 ± 0.23 ^a^	0.999 ± 0.000	1.56 ± 0.30 ^a^	0.027 ± 0.002 ^a^	36.89 ± 2.11 ^a^	0.992 ±0.009
Dydimin	0.726 ± 0.321 ^a^	0.022 ± 0.000 ^a^	44.79 ± 0.96 ^b^	0.997 ± 0.002	0.90 ± 0.17 ^a^	0.018 ± 0.001 ^b^	55.29 ± 1.88 ^a^	0.998 ±0.001
Apigenin 6,8-di-C-glucoside	0.814 ± 0.113 ^a^	0.010 ± 0.002 ^a^	102.63 ± 19.34 ^a^	0.984 ± 0.021	1.70 ± 0.62 ^a^	0.011 ± 0.000 ^a^	87.80 ± 3.29 ^b^	0.972 ±0.029
Total, Flavanones	0.411 ± 0.107 ^b^	0.020 ± 0.000 ^a^	50.08 ± 0.15 ^b^	1.000 ± 0.000	0.96 ± 0.24 ^a^	0.018 ± 0.001 ^b^	56.27 ± 2.68 ^a^	0.996 ±0.003
Total, Flavones	1.437 ± 0.213 ^a^	0.017 ± 0.004 ^a^	58.98 ± 11.14 ^a^	0.983 ± 0.021	3.09 ± 1.23 ^a^	0.021 ± 0.001 ^a^	47.29 ± 1.78 ^b^	0.972 ±0.029
Total (poly)phenols	0.491 ± 0.071 ^b^	0.020 ± 0.000 ^a^	50.26 ± 0.90 ^b^	0.999 ± 0.000	1.05 ± 0.25 ^a^	0.018 ± 0.001 ^b^	55.33 ± 2.59 ^a^	0.996 ±0.003

Values following by the same letter, in the same row, are not significantly different (*p* > 0.05) according to Tukey’s Multiple Range Test.

## Data Availability

Data will be made available on request.

## Supplementary Materials

The following supporting information can be downloaded at https://www.mdpi.com/article/10.3390/polym18202533/s1, Table S1: Reference standards used for HPLC identification and quantification, grouped by compound class; Table S2: Recovery of polyphenol compounds of chitosan/gelatin film added with orange peel extract (CGF-Ex) and chitosan/gelatin film added with orange peel extract loaded in nanoemulsion (CGF-N); Table S3: Weibull model parameters (β, τ, R^2^) for phenolic compounds release from CGF-Ex and CGF-N.
