# Supplementary material for: Optimization of Orange Peel Extract Nanoemulsions and Their Incorporation into Chitosan/Gelatin Biodegradable Films: Physicochemical and Functional Properties
Source: Polymers (Basel). 2026 Oct 15;18(20):2533. doi: 10.3390/polym18202533 (PMC13708873; doi:10.3390/polym18202533)
Supplement: Supplementary file 1 [file polymers-18-02533-s001.zip › polymers-4596023-supplementary.pdf]

Supplementary Table S1. Reference standards used for HPLC identification and quantification, grouped by compound class.

Subfamily	Compounds
**Hydroxybenzoic acids**	Gallic acid, Protocatechuic acid, 4-Hydroxybenzoic acid, Vanillic acid, Ellagic acid, Benzoic acid, Syringic acid, Vanillin
**Hydroxycinnamic acids**	Chlorogenic acid, Caffeic acid, *p*-Coumaric acid, Sinapic acid, Ferulic acid, Rosmarinic acid
**Flavanones**	Eriocitrin, Narirutin, Naringin, Hesperidin, Dydymin, Poncirin, Eriodicytol, Narigenin, Hesperetin,
**Flavones**	Isoorientin, Vitexin-4-O-glucose, Vitexin-2-O-rhamnose, Vitexin, Luteolin 7-O-β-D-glucoside, Diosmin, Apigenin 7-glucoside, Neodiosmin, Luteolin, Apigenin, Chysoeriol
**Flavonols**	Rutin, Hyperoside, Quercetin-3-β-glucose, Isorhamnetin-3-o-glucoside, Kaempherol-3-glucoside, Quercitrin, Myricetin, Quercetin, Kaempferol, Rhamnetin
**Flavan-3-ols and gallates**	Catechin, Epicatechin, Gallocatechin gallate, Epigallocatechin-3-gallate (MIX), Gallocatechin-3-gallate (MIX), Epicatechin-3-gallate (MIX), Catechin-3-gallate (MIX)
**Proanthocyanidins**	Proanthocyanidin A2, Proanthocyanidin B2
**Anthocyanins and anthocyanidins**	Pelargonidin 3-O-β-glucopyranoside (MIX), Malvidin-3-5-O-di-β-glucopyranoside, Cyanidin-3-glucopyranoside (MIX), Peonidin 3-O-β-glucopyranoside (MIX), Delphinidin 3-O-β-glucopyranoside (MIX), Petunidin 3-O-β-glucopyranoside (MIX), Malvidin-3-O-glucopyranoside, Malvidin
**Ellagitannins**	Punicalagin A, Punicalagin B
**Phenylethanoids**	Hydroxytyrosol, Tyrosol, Verbascoside
**Others**	Caffeine (alkaloid), L-Tryptophan (amino acid), Oleuropein (secoiridoid)

Supplementary Table S2. Recovery of polyphenol compounds of chitosan/gelatin film added with orange peel extract (CGF-Ex) and chitosan/gelatin film added with orange peel extract loaded in nanoemulsion (CGF-N)

	CGF-Ex	CGF-N
**Phenolic acids**		
Ferulic acid glycoside	14.81 ± 0.95^{b}	35.79 ± 4.46^{a}
Feruloylcoumarate	24.60 ± 1.30^{a}	11.04 ± 1.40^{b}
**Flavanones**		
Naringenin triglycoside	65.18 ± 7.06^{a}	72.40 ± 2.88^{a}
Hesperitin tryglicoside	52.37 ± 7.88^{b}	148.93 ± 11.45^{a}
Narirutin	61.24 ± 10.28^{a}	64.08 ± 1.15^{a}
Hesperidin	86.10 ± 7.36^{b}	101.51 ± 3.95^{a}
Dydimin	79.35 ± 2.43^{a}	80.50 ± 5.94^{a}
**Flavones**		
Diosmetin 6,8-di-C-glucoside	39.92 ± 3.19^{a}	58.69 ± 12.18^{a}
Diosmin	59.34 ± 4.14^{a}	72.77 ± 6.65^{a}
Diosmetin glycoside	59.15 ± 1.35^{b}	78.53 ± 3.35^{a}
Apigenin 6,8-di-C-glucoside	49.22 ± 7.04^{a}	59.23 ± 2.35^{a}
Vitexin-2-O-rhamnose	39.53 ± 1.41^{b}	54.93 ± 2.88^{a}
Vitexin	29.14 ± 1.83^{b}	67.68 ± 7.29^{a}
Total (poly)phenols	66.82 ± 0.78^{b}	83.59 ± 1.98^{a}
Toral Phenolic acids	7.06 ± 0.23^{b}	16.01 ± 1.90^{a}
Total Flavones	40.79 ± 3.73^{b}	60.35 ± 3.15^{a}
Total Flavanones	75.99 ± 1.34^{b}	88.42 ± 2.55^{a}

Values are expressed as mean ± SD. Different superscript letters (a, b) in the same row indicate statistically significant differences according to Tukey's test ($p < 0.05$).

Supplementary Table S3. Weibull model parameters (β, τ, R^2) for phenolic compounds released from CGF-Ex and CGF-N.

	CGF-Ex			CGF-N		
	β	τ (min)	R^2	β	τ (min)	R^2
Naringenin triglycoside	0.81 ± 0.14^a	31.20 ± 2.54^a	0.869 ± 0.050	0.76 ± 0.13^a	42.62 ± 3.25^b	0.969 ± 0.007
Hesperitin tryglicoside	1.28 ± 0.46^a	27.09 ± 1.84^a	0.951 ± 0.058	0.50 ± 0.06^b	57.89 ± 12.00^b	0.935 ± 0.008
Narirutin	1.37 ± 0.33^a	34.02 ± 1.86^a	0.920 ± 0.064	0.54 ± 0.12^b	93.50 ± 22.01^b	0.893 ± 0.057
Hesperidin	1.39 ± 0.32^a	33.25 ± 4.24^a	0.858 ± 0.127	0.69 ± 0.16^b	75.14 ± 18.93^b	0.928 ± 0.037
Apigenin 6,8-di-C-glucoside	0.38 ± 0.11^a	258.66 ± 77.92^a	0.847 ± 0.104	0.55 ± 0.03^a	145.81 ± 47.02^a	0.891 ± 0.055
Total Flavanones	1.35 ± 0.15^a	34.73 ± 2.92^a	0.900 ± 0.095	0.56 ± 0.11^b	87.13 ± 22.87^b	0.927 ± 0.050
Total polyphenols	1.28 ± 0.12^a	35.76 ± 2.34^a	0.910 ± 0.080	0.65 ± 0.12^b	83.24 ± 18.30^b	0.947 ± 0.025

CGF-Ex: Chitosan/gelatin film added with orange peel extract; CGF-N: Chitosan/gelatin film added with orange peel extract loaded in nanoemulsion. Values are expressed as mean ± SD. Different superscript letters (a, b) in the same row and for the same kinetic parameter indicate statistically significant differences between CGF-Ex and CGF-N according to Tukey's test ($p < 0.05$).
